# Hereditary determinants of gynecological cancer and recommendations: Number 8 - August 2021

**DOI:** 10.1055/s-0041-1736211

**Published:** 2021-09-21

**Authors:** Jesus Paula Carvalho, Filomena Marino Carvalho, Anisse Marques Chami, Agnaldo Lopes da Silva Filho, Walquíria Quida Salles Pereira Primo

**Affiliations:** 1 Universidade de São Paulo, São Paulo, SP, Brazil; 2 Universidade de São Paulo, São Paulo, SP, Brazil; 3 Universidade Federal de Minas Gerais, Belo Horizonte, MG, Brazil; 4 Universidade Federal de Minas Gerais, Belo Horizonte, MG, Brazil; 5 Universidade de Brasília, Brasília, DF, Brazil

## Key points

- Hereditary genetic factors are responsible for a considerable part of gynecological malignancies.
- Knowing these factors enables precision prevention.
- Non-HPV-associated cervical adenocarcinomas (NHPVA) are diseases related to hereditary genetic factors.
- Among endometrial carcinomas, 3%-5% are hereditary (Lynch syndrome) and in half of cases they precede colorectal cancer, which is the most detected.
- Ovarian, tubal and peritoneal high-grade serous carcinomas originate in the epithelium of the tubal fimbriae.
- Approximately 20% of ovarian carcinomas are hereditary.
- Pathogenic mutations of BRCA1 and BRCA2 genes are more frequent in ovarian cancer, but other genes are also related to this disease.
- The identification of individuals at hereditary risk for gynecological cancer and prophylaxis are the most efficient measures.
- Assessment of family history and appropriate genetic tests interpreted by qualified professionals are cost-effective.
- Clinical therapeutic measures and prophylactic surgeries are part of the resources for prevention of hereditary gynecological cancer.

## Recommendations

- Although rare, there is an association of germline mutations as a predisposing factor to cervical cancer. A genetic investigation is justified when the family context and the histological type of the neoplasm suggest an association with predisposing genes or syndromes.
- Endometrial cancer should be thought of as an important finding for the differential diagnosis of hereditary cancer predisposition syndrome.
- Immunohistochemical panel investigation for DNA repair proteins in endometrial cancer may be important for the exclusion of Lynch syndrome, regardless of age at diagnosis.
- The diagnosis of ovarian cancer, mainly high-grade serous, itself, is a criterion for the study of possible germline mutations associated with a higher risk for this type of cancer.
- Specific knowledge is necessary for the interpretation of genetic tests in the face of results that may be informative or not to guide medical conduct.
- Genetic counseling should be indicated whenever possible in cases of suspected or diagnosed germline mutation and/or syndromes associated with the risk for hereditary cancer.

## Clinical context

Hereditary genetic factors are responsible for a considerable portion of gynecological malignancies, especially ovarian and endometrial cancer, and in a very small number, although not negligible, of cervical cancer. 1 People who have not had cancer, but are more likely than the rest of the population to have it, are called previvors. 2 These people demand different care and interventions from routine measures. Many women seen by gynecologists have cancer susceptibility genes (CSG) and may develop some type of gynecological cancer. Identifying these women and taking appropriate measures so that they do not develop the disease is part of the mission of gynecologists.

The identification of women with CSG may allow treatments with target therapies in already sick patients. However, the greatest benefit is in what is called “precision prevention”, which is a strategy that incorporates actio ns on environmental, epidemiological, hormonal, lifestyle and behavioral factors. The knowledge of genetic determinants of diseases allows the use of information from carriers of mutations to guide tests in their close relatives. In this text, we will address only the hereditary determinants of cervical, endometrial, ovarian, tubal and peritoneal cancers. The subject is much broader. Women with these types of cancer are often at risk for malignant neoplasms in other organs. This is just the beginning of a wide discussion.

## Is cervical cancer associated with any inherited genetic risk factor?

Human papillomavirus (HPV) is responsible for 99.7% of malignant cervical tumors. 3 Squamous cell carcinomas represent 60% to 80% of these tumors and adenocarcinomas, 10% to 20%. The other carcinomas represent 1.1% to 6.1%, and sarcomas, less than 1%.

In the group of cervical adenocarcinomas, 10%-15% of cases are classified as non-HPV associated (NHPVA). 4 These NHPVA tumors are usually p16 negative and p53 overexpression. Gastric-type adenocarcinoma is a variant of endocervical mucinous adenocarcinoma that is non-HPV-associated and exhibits aggressive behavior and chemoresistance. It also has genetic similarities with pancreatobiliary carcinoma. The most frequently mutated genes in gastric endocervical adenocarcinoma are TP53 (52.4%), STK11, HLA-B and PTPRS (19.0%), FGFR4 (14.3%), GNAS, BRCA2, ELF3, ERBB3, KMT2D and SLX4 (9.5%), CDH1, EPCAM, KRAS, MLH1, RNF43, SNAI1, TWIST1, ZEB1 and ZEB2 (1/21, 4.8%). 5

Gastric-type adenocarcinomas are related to Peutz-Jeghers syndrome. 6 Women with mutations in MMR genes (DNA repair genes) have an estimated 5.6 times greater risk for cervical cancer than the general population. 7 The definition of the site of origin of cervical adenocarcinoma in women with Lynch syndrome (LS) may be difficult, as this syndrome is also associated with endometrium carcinoma of the lower uterine segment. 8 Minimal deviation adenocarcinoma is a morphologically well differentiated variant of gastric-type adenocarcinoma. Despite its apparently benign morphology, it has aggressive behavior and poor prognosis.

Small cell neuroendocrine carcinoma of the cervix is a rare tumor and may be related to HPV, especially HPV 18, which presents loss of mismatch repair (MMR) enzyme expression in 33% of cases. 9

In a study of first-degree relatives of women with invasive cervical carcinoma in Latin America and a control group, was found no difference in the incidence of cervical cancer in first-degree relatives. 10

## Which hereditary cancer predisposition syndromes are associated with endometrial cancer risk and which clinical aspects are relevant to recognize them?

Germline mutations in CSGs are responsible for 3% of endometrial carcinoma and 5% in women under 70 years of age.

### Lynch syndrome

Lynch syndrome is caused by germline mutations in the DNA mismatch repair genes (MMR genes) MLH1, MSH2, MSH6 and PMS2 or deletion of the EPCAM gene (epithelial cell adhesion molecule), which causes MSH2 inactivation. 11 Women with LS have 43% to 57% risk for endometrial carcinoma during their lifetime. 12 An individual is generally considered to have LS if they have a pathogenic variant in one of the MMR genes, regardless of having been diagnosed with an associated cancer. 13

The younger the patient with endometrial carcinoma, the more likely she is to have LS. In 35 consecutive patients with endometrial carcinoma and less than 50 years of age, LS was diagnosed in 22.8% of cases by clinical and/or molecular criteria. 14 In addition to endometrial carcinoma, these women are at higher risk for other types of cancer such as colorectal, ovarian, stomach cancer, among others. 15

In the United Kingdom, a project was developed to test patients with endometrial carcinoma by means of immunohistochemistry to detect deficiency of MMR genes, test for hypermethylation of the MLH1 promoter and genetic test for pathogenic variants of MMR. Testing for LS in all patients with endometrial carcinoma proved to be cost-effective. 13

Mutations of DNA mismatch repair genes (MMR) cause variations in microsatellites and changes in the length of the genome, leading to the event known as microsatellite instability (MSI), which is a hallmark of LS. 11

Thus, the analysis of proteins corresponding to the DNA repair genes by immunohistochemistry, or even the search for MSI by molecular methods are important to guide the diagnosis of LS in the evaluation of patients with suspicion. In addition, as it is an autosomal dominant hereditary syndrome, family history is essential for high suspicion of LS. The revised Amsterdam II criteria, for example, guide clinical diagnosis and are determined by all of the following characteristics: 16

- Three or more relatives affected by cancer of the LS spectrum (colorectal, endometrial, small intestine, stomach, ureter or renal pelvis, biliary tract, brain, skin [sebaceous tumor]);
- An individual must be a first-degree relative of the other two;
- Two or more successive generations affected;
- One or more relatives must have been diagnosed with cancer before the age of 50;
- Familial adenomatous polyposis must have been excluded in cases of colorectal cancer;
- All tumors must be checked by anatomopathological examination.

### PTEN gene mutation associated with Cowden syndrome and endometrial cancer

This syndrome affects 1 in 200,000 people and is associated with a predisposition to breast, thyroid, kidney, endometrial and colon cancer and melanoma. 17 Endometrial carcinoma occurs in 21%-28% of these women and usually develops at very early ages. 18 Although it is a very rare syndrome, the diagnostic criteria are clinically recognizable and the patient usually presents typical morphological findings: macrocrania (percentile greater than 97), facial tricholemomas, oral papillomas and palmoplantar keratosis. Endometrial cancer, as well as breast cancer, follicular thyroid cancer and macrocrania, are major criteria for Cowden syndrome. 19

### Mutations in the BRCA1 and BRCA2 genes and endometrial cancer

A 2002 study of 11,847 women with a BRCA1 gene mutation demonstrated a two to three-fold higher risk for endometrial carcinoma, although many of these women were users of tamoxifen for treatment and/or prophylaxis of breast cancer, and tamoxifen alone could be a risk factor for endometrial carcinoma. 20 In another 2020 study of 1,350 BRCA1 and 1,259 mutated BRCA2, a higher risk for endometrial carcinoma was not identified, not even for serous carcinoma. 21

## When to indicate endometrial cancer risk reduction surgery?

An analysis of data from 18 countries from the “Prospective Lynch Syndrome Database (PLSD)” 22 demonstrated that 95% of centers offer total hysterectomy and bilateral salpingo-oophorectomy (TH + BSO) for patients with deleterious mutations in MLH1 and MSH2, 91% in MSH6 and 67% in PSM2. Hormone therapy with isolated estrogens is offered to 71% of women aged 35 to 55 years. In addition to estrogen, protection against colorectal cancer must be offered. 22

In women with deleterious mutations in BRCA genes, so far, there are no formal recommendations to remove the uterus together with the risk-reducing BSO (rrBSO), but the relationship between hereditary mutation in the BRCA1 gene and the development of endometrial serous carcinoma has been proposed by several authors. 16 Hysterectomy together with BSO can be an option to be considered, given the need for hormone replacement. The potential risk of endometrial cancer should be considered and discussed with the patient, in relation to the advantages and the risk of also having a hysterectomy at the time of the risk-reducing surgery for ovarian cancer in women with a deleterious mutation of the BRCA1 gene. 23

Women with deleterious mutations in the BRCA1 or BRCA2 genes undergoing prophylactic BSO may experience improved menopausal symptoms with hormone replacement. Mejia-Gomez et al. 24 found that only 61% of these women under the age of 50 underwent hormone therapy. Estrogenic therapy in young women reduces the risk of vulvovaginal atrophy, osteoporosis, dyspareunia, atherosclerosis, cardiovascular disease and possibly dementia. 24 In women with an intact uterus, adding progestogens to hormone therapy is recommended to prevent hyperplasia and endometrial carcinoma. 25 However, the use of progestogens has been associated with a higher risk of breast cancer in women with a BRCA1 mutation. 26

For women with Cowden syndrome, the National Comprehensive Cancer Network® 27 recommends starting screening at age 35. Women with a mutation should be instructed to recognize early symptoms of endometrial carcinoma, such as vaginal bleeding. Endometrial biopsy screening should be considered at one to two years intervals. Transvaginal ultrasound is not recommended in pre-menopause, considering the variation in endometrial thickness. Hysterectomy should be considered as soon as the patient has completed the pregnancies.

## How to deal with the risk of ovarian cancer (including uterine tubes and peritoneum) in view of the considerable possibility of the inherited genetic etiology associated with this type of cancer?

Deleterious germline mutations result in loss of function in different genes related to a higher risk of ovarian cancer and breast cancer. In this section, we will restrict the analysis to the risk of ovarian, tubal and peritoneal cancer, which is currently classified as a single entity, although in this text we will call it only ovarian cancer. The main genes whose loss of function are related to ovarian cancer are: BRCA1, BRCA2, BARD1, BRIP1, CHEK2, MRE11A, MSH6, MLH1, MSH2, NBN, PALB2, RAD50, RAD51C and TP53, and the BRCA1 and BRCA2 genes are the most frequently involved, with 40% and 23%, respectively, of cases of hereditary ovarian, tubal and peritoneal cancer. 19

The BRCA1 and BRCA2 are tumor suppressor genes that repair breaks in the double strand of DNA to maintain genomic stability in a process called homologous recombination repair (HRR). 28

Approximately 20% of high-grade ovarian serous carcinomas have germline mutations in the BRCA1 and BRCA2 genes, and 95% of these tumors have somatic mutations in the TP53 genes with loss of p53 protein function. 29 Germline mutations in the BRCA1 and BRCA2 genes predispose to the development of somatic mutations in the TP53 genes. The risk of developing ovarian cancer varies between 39%-63% and 16.5%-27% in women with pathogenic mutations in the BRCA1 and BRCA2, respectively. 28

Ovarian, tubal and peritoneal high-grade serous carcinoma (HGSC) develops from precursor tubal lesions, that is, serous tubal intraepithelial cancer (STIC), which is estimated to precede the appearance of invasive carcinoma by seven years. 30 Mutational analyzes revealed identical TP53 gene mutations in STICs and serous carcinomas, suggesting a common monoclonal origin. 31

Another precursor lesion even earlier than STIC is the p53 signature lesion (p53 signaling pathway), characterized by the growth of cells in the distal portion of the tubal fimbria that shares properties with serous ovarian cancer - including p53 mutations - and is a precursor to HGSC. 32 The p53 signature are extremely small lesions represented by 10-30 cells. Histological and immunohistochemical (p53) exams in uterine tube specimens from patients with ovarian HGSC demonstrated p53 signature in 17.9% and STICs in 6.5% of specimens. 31

The natural history of HGSC starts in the secretory cell of the fimbriae that acquires the TP53 gene mutation, evolves into the precursor lesion called “p53 signature”, which is not identified in a conventional anatomopathological examination of hematoxylin and eosin (HE) and requires immunohistochemical examination. Then, it evolves into STIC, which can already be detected in conventional HE exams and later turns into HGSC, which affects the ovaries and peritoneum, already as a metastatic tumor. 2 33

The process begins at the first ovulation and develops over 30 years, with ten years elapsing from the normal tubal epithelium until the p53 signature, another fifteen years to evolve to the intraepithelial tubal neoplasia, and finally, another five years to the HGSC. 34

### Investigate family history of cancer

Understanding the natural history and pathogenesis of ovarian, tubal and peritoneum HGSC is the key to plan detection and prevention strategies. This in itself would prevent the inappropriate use of resources in imaging exams, tumor markers and other markers for the purpose of screening and early diagnosis of ovarian cancer that have proven ineffective in several studies. 35 Family history of cancer, and not just ovarian and breast cancer, should be valued and investigated with rigor and detail.

### Genetic testing

For women with histories suggestive of family cancer, it is recommended to offer specific genetic tests and evaluation, preferably with the guidance of a trained professional to provide genetic counseling. In the United Kingdom, genetic tests are offered for individuals with a family history indicating at least 10% probability of carrying mutations in BRCA genes. 1

The use of multigenic panels has become common in clinical practice, even though the tested genes vary significantly in different commercial tests and can lead to confusion or misinterpretation of results. 36 More important than asking for genetic tests, is knowing how to interpret them properly. Misinformation can be even more damaging.

### Risk-reducing bilateral salpingo-oophorectomy (rrBSO)

Risk-reducing bilateral salpingo-oophorectomy is the most effective strategy and also the gold standard for reducing the risk of ovarian cancer in high-risk women. 37 The acceptance of BSO is difficult for women in the 35-40 age group, given the effects on hormonal, sexual, reproductive and emotional function, osteoporosis and premature death, even if this intervention is effective in reducing the risk of death from ovarian cancer. 38 On the other hand, there are still few centers offering prophylactic surgeries for ovarian cancer as a routine. 39

Another source of resistance to rrBSO are doctors themselves. Even with all the evidence of the benefits of this surgery in high-risk women, there is a considerable number of gynecologists who resist to indicate prophylactic surgeries in women of proven risk. 40 However, as genetic assessments and tests become more used, they are likely to increase acceptance and availability of procedures. In any case, it is important that this is done in reference centers and by competent specialists to evaluate clinical data and genetic tests. 40 In Brazil, at present, it is still difficult to include genetic testing and prophylactic procedures in clinical practice, but this demands work until becoming a reality, including in the National Health Service (Brazilian SUS). 41

### Early risk-reducing bilateral salpingectomy and late bilateral oophorectomy

An alternative that is being tested to circumvent these problems is early salpingectomy and late oophorectomy. Ovarian carcinoma originates in the tubes and only later spreads to the ovaries. Thus, the earlier the tubes are removed, the greater the chance of avoiding ovarian contamination by tubal neoplastic cells or, more likely, by tubular cells that are still normal, but will become neoplastic when implanted in the ovaries. There are no conclusive studies demonstrating the effectiveness of this strategy, but studies are underway to test this hypothesis. 37

## Final considerations

Genetic counseling can be an important tool in the clinical evaluation of women with gynecological cancer. Family history, as well as the age of diagnosis below 50 years are facts that suggest the possibility of hereditary cancer. However, for ovarian cancer of epithelial origin, regardless of age at diagnosis and family history, counseling should be offered to women for assessment of the differential diagnosis of family cancer in view of the high frequency of germline mutations associated with the risk for this cancer. Identifying the hereditary pattern of cancer is essential, as it allows guiding cancer risk reduction strategies for patients and their family members.

National Specialty Commission for Gynecologic Oncology of the Brazilian Federation of Gynecology and Obstetrics Associations (FEBRASGO)

President:

Walquíria Quida Salles Pereira Primo

Vice-President:

Suzana Arenhart Pessini

Secretary:

Jesus Paula Carvalho

Members:

Angélica Nogueira Rodrigues

Caetano da Silva Cardial

Delzio Salgado Bicalho

Eduardo Batista Candido

Etelvino de Souza Trindade

Fernando Maluf

Francisco José Cândido dos Reis

Filomena Marino Carvalho

Georgia Fontes Cintra

Marcia Luiza Appel Binda

Mirian Helena Hoeschl Abreu Macedo

Renato Moretti Marques

Ricardo dos Reis

Sophie Françoise Mauricette Derchain

Heloisa de Andrade Carvalho
